# Genetic characterization of caffeine degradation by bacteria and its potential applications

**DOI:** 10.1111/1751-7915.12262

**Published:** 2015-02-12

**Authors:** Ryan M Summers, Sujit K Mohanty, Sridhar Gopishetty, Mani Subramanian

**Affiliations:** 1 Department of Chemical and Biological Engineering, The University of AlabamaTuscaloosa, AL, 35487, USA; 2 Department of Chemical and Biochemical Engineering, The University of IowaCoralville, IA, 52241, USA; 3 Center for Biocatalysis and Bioprocessing, The University of IowaCoralville, IA, 52241, USA

## Abstract

The ability of bacteria to grow on caffeine as sole carbon and nitrogen source has been known for over 40 years. Extensive research into this subject has revealed two distinct pathways, *N*-demethylation and C-8 oxidation, for bacterial caffeine degradation. However, the enzymological and genetic basis for bacterial caffeine degradation has only recently been discovered. This review article discusses the recent discoveries of the genes responsible for both *N*-demethylation and C-8 oxidation. All of the genes for the *N*-demethylation pathway, encoding enzymes in the Rieske oxygenase family, reside on 13.2-kb genomic DNA fragment found in *Pseudomonas putida* CBB5. A nearly identical DNA fragment, with homologous genes in similar orientation, is found in *Pseudomonas* sp. CES. Similarly, genes for C-8 oxidation of caffeine have been located on a 25.2-kb genomic DNA fragment of *Pseudomonas* sp. CBB1. The C-8 oxidation genes encode enzymes similar to those found in the uric acid metabolic pathway of *Klebsiella pneumoniae*. Various biotechnological applications of these genes responsible for bacterial caffeine degradation, including bio-decaffeination, remediation of caffeine-contaminated environments, production of chemical and fuels and development of diagnostic tests have also been demonstrated.

## Introduction

Caffeine (1,3,7-trimethylxanthine) and related methylxanthines are natural purine alkaloids found in many plants around the world (Ashihara and Crozier, 1999). These compounds are hypothesized to serve as natural insecticides, and have been shown to protect the plants from insects and other predators (Nathanson, 1984; Hollingsworth *et al*., 2002). Other possible reasons for biosynthesis of caffeine include inhibition of plant matter (Waller, 1989) and improved pollination (Wright *et al*., 2013).

Methylxanthines are often consumed by humans in foods and beverages, including chocolate, coffee and tea. Coffee is a major worldwide agricultural commodity, with millions of metric tons produced and distributed globally each year (Summers *et al*., 2014). In addition to the food industry, caffeine and related methylxanthines are also used in pharmaceuticals as stimulants, diuretics, bronchodilators, vasodilators and in the treatment and/or prevention of axial myopia, glaucoma and macular degeneration (Stavric, 1988a,b,c; Trier *et al*., 1999; Dash and Gummadi, 2006b; Daly, 2003).

Although bacterial caffeine degradation has been studied since the 1970s, very little was known concerning the enzymes and genes responsible for caffeine degradation until recently. Several excellent reviews have summarized the bacterial caffeine catabolic pathways (Mazzafera, 2004; Dash and Gummadi, 2006a,b; Gummadi *et al*., 2012), including recent developments (Gopishetty *et al*., 2012). In this review, we focus on the discoveries made since the publication of these reviews, with specific emphasis on the genes responsible for caffeine degradation in bacteria.

## Caffeine metabolism

To date, over 35 bacterial strains that are capable of degrading caffeine have been isolated and reported (Table 1). While there is some diversity among the types of bacteria isolated, the majority are *Pseudomonas*, primarily *Pseudomonas putida*. Caffeine-degrading bacteria are geographically dispersed, and have been found in coffee fields (Yamaoka-Yano and Mazzafera, 1998), wastewater streams (Ogunseitan, 1996) and garden soil (Blecher and Lingens, 1977; Yu *et al*., 2008; 2009; 2014).

**Table 1:** Characterized bacterial strains capable of degrading caffeine

Organism	Location isolated	Catabolic pathway	References
*Pseudomonas putida* strain 40	California, USA	*N*-demethylation	Woolfolk, 1975
*Pseudomonas putida* C1	Germany	*N*-demethylation	Blecher and Lingens, 1977
*Pseudomonas putida* C3024	Netherlands	N.R.	Middelhoven and Bakker, 1982
*Pseudomonas putida* WS	Germany	*N*-demethylation	Glück and Lingens, 1987
*Pseudomonas* sp. No. 6	Japan	*N*-demethylation	Asano *et al*., 1993
*Pseudomonas putida* No. 352	Japan	*N*-demethylation	Asano *et al*., 1994
*Serratia marcescens*	Brazil	*N*-demethylation	Mazzafera *et al*., 1996
*Pseudomonas putida* ATCC 700097	California, USA	*N*-demethylation	Ogunseitan, 1996
*Klebsiella* and *Rhodococcus*	India	C-8 oxidation	Madyastha and Sridhar, 1998
*Pseudomonas putida* (8 strains)	Brazil	N.R.	Yamaoka-Yano and Mazzafera, 1998
*Pseudomonas fluorescens*	Brazil	N.R.	Yamaoka-Yano and Mazzafera, 1998
Coryneform (4 strains)	Brazil	N.R.	Yamaoka-Yano and Mazzafera, 1998
*Acinetobacter* sp. (3 strains)	Brazil	N.R.	Yamaoka-Yano and Mazzafera, 1998
*Flavobacterium* sp. (2 strains)	Brazil	N.R.	Yamaoka-Yano and Mazzafera, 1998
*Moraxella* sp.	Brazil	N.R.	Yamaoka-Yano and Mazzafera, 1998
*Pseudomonas putida* IF-3	Japan	*N*-demethylation	Koide *et al*., 1996
*Pseudomonas putida* L	Brazil	*N*-demethylation	Yamaoka-Yano and Mazzafera, 1999
*Pseudomonas putida* KD6	N.R.	*N*-demethylation	Sideso *et al*., 2001
*Alcaligenes* sp.	Canada	C-8 oxidation	Mohapatra *et al*., 2006
*Pseudomonas putida* NCIM 5235	India	*N*-demethylation	Dash and Gummadi, 2006a
*Pseudomonas* sp. CBB1	Iowa, USA	C8-oxidation	Yu *et al*., 2008
*Pseudomonas alcaligenes* CFR 1708	India	N.R.	Sarath Babu *et al*., 2005
*Alcaligenes fecalis* T1	India	N.R.	Sarath Babu *et al*., 2005
*Acetobacter* sp. T3	India	N.R.	Sarath Babu *et al*., 2005
*Pseudomonas putida* CBB5	Iowa, USA	*N*-demethylation	Yu *et al*., 2009
*Pseudomonas* sp. CES	Iowa, USA	*N*-demethylation	Yu *et al*., 2014

N.R., not reported.

Metabolic studies with these caffeine-degrading bacterial isolates have revealed only two catabolic pathways: *N*-demethylation and C-8 oxidation. The *N*-demethylation pathway appears to be the most common, as it has been observed in over 80% of reported isolates where metabolism has been characterized. In both pathways, bacteria break caffeine down to carbon dioxide and ammonia to harvest energy and cellular building blocks.

**Fig 1:**
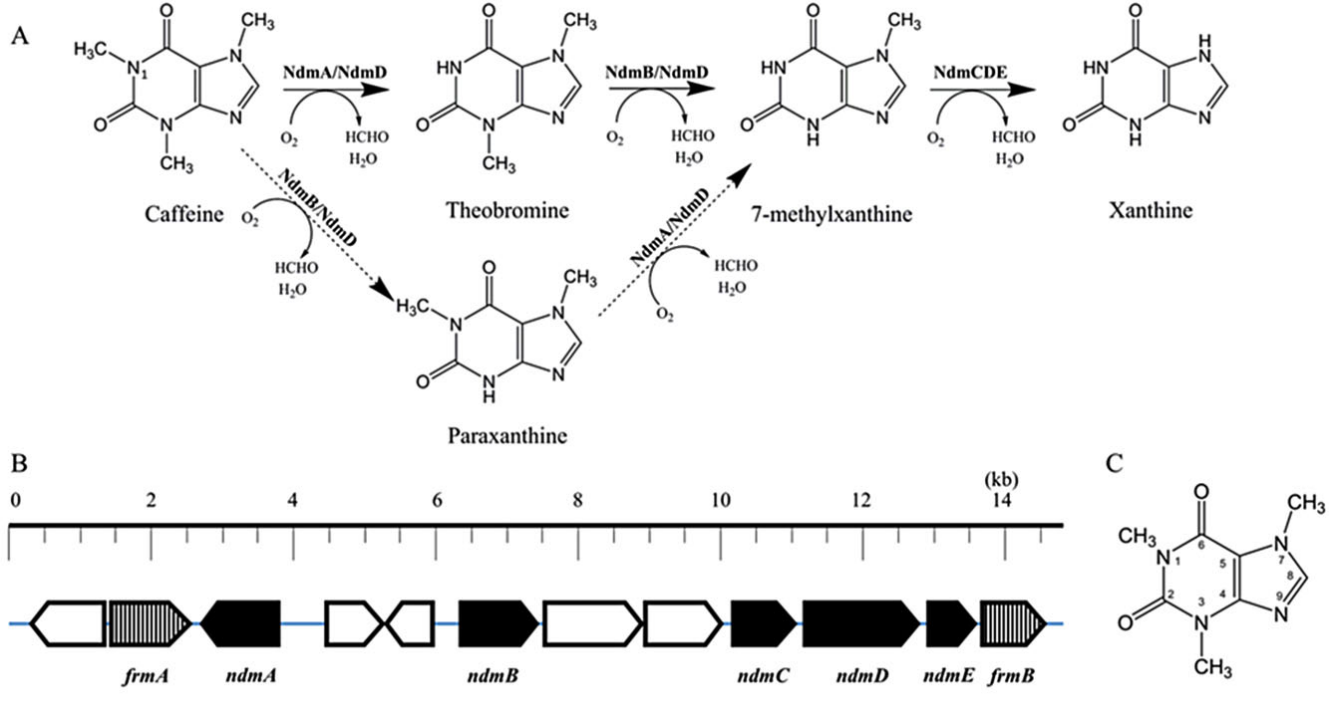
Proposed caffeine *N*-demethylation pathway (A) and map of associated genes (B) in *Pseudomonas putida* CBB5. The dashed arrows in part (A) represent a minor pathway, accounting for 1–2% of metabolized caffeine. NdmA/*ndmA* = *N*_1_-demethylase specific for *N*_1_-methyl group of caffeine; NdmD/*ndmD* = reductase; NdmB/*ndmB* = *N*_3_-demethylase specific for *N*_3_-methyl group of theobromine; NdmCDE = protein complex containing *N*_7_-demethylase specific for *N*_7_-demethylation of 7-methylxanthine; *ndmC* = NdmC gene; *ndmE* = NdmE gene; *frmA* = glutathione-dependent formaldehyde dehydrogenase; *frmB* = *S*-formylglutathione hydrolase. (C) A numbered structure of the caffeine molecule.

During *N*-demethylation, the caffeine molecule is sequentially *N*-demethylated to form xanthine (Fig. 1A). Each of the three methyl groups is removed with incorporation of molecular oxygen to produce one formaldehyde and one water molecule per reaction. Theobromine (3,7-dimethylxanthine) is the major metabolite formed from the first step in the pathway, with small amounts of paraxanthine (1,7-demethylxanthine) also reported in some strains (Yamaoka-Yano and Mazzafera, 1999; Yu *et al*., 2009; 2014). The second step of the pathway is the *N*_3_-demethylation of theobromine or the *N*_1_-demethylation of paraxanthine to form 7-methylxanthine. 7-Methylxathine is further *N*_7_-demethylated to form xanthine. Finally, xanthine is converted to uric acid, which enters normal purine catabolic pathway.

Theophylline (1,3-dimethylxanthine) has not been reported as a metabolite of caffeine in bacteria. However, it is the first major metabolite of caffeine in fungi (Hakil *et al*., 1998), and is further degraded via *N*-demethylation to 3-methylxanthine and xanthine. Although the bacterium *P. putida* CBB5 does not produce theophylline from caffeine, it has been reported to degrade theophylline by *N*-demethylation (Yu *et al*., 2009). Both 3-methylxanthine (major product) and 1-methylxanthine (minor product) are formed from theophylline in CBB5 and are further *N*-demethylated to form xanthine, as in the caffeine catabolic pathway.

Some of the metabolites formed during the bacterial *N*-demethylation of caffeine also undergo C-8 oxidation to form their corresponding uric acids (Blecher and Lingens, 1977; Yamaoka-Yano and Mazzafera, 1999). In most strains, this involves the formation of 3,7-dimethyluric acid, 1,7-dimethyluric acid and 7-methyluric acid from theobromine, paraxanthine and 7-methylxanthine respectively. These methyluric acids are not formed during caffeine *N*-demethylation in *P. putida* CBB5. However, CBB5 converts approximately 25% of the entire theophylline metabolite pool to 1,3-dimethyluric acid, 1-methyluric acid and 3-methyluric acid. There is no evidence that these methyluric acids are further metabolized (Yu *et al*., 2009).

**Fig 2:**
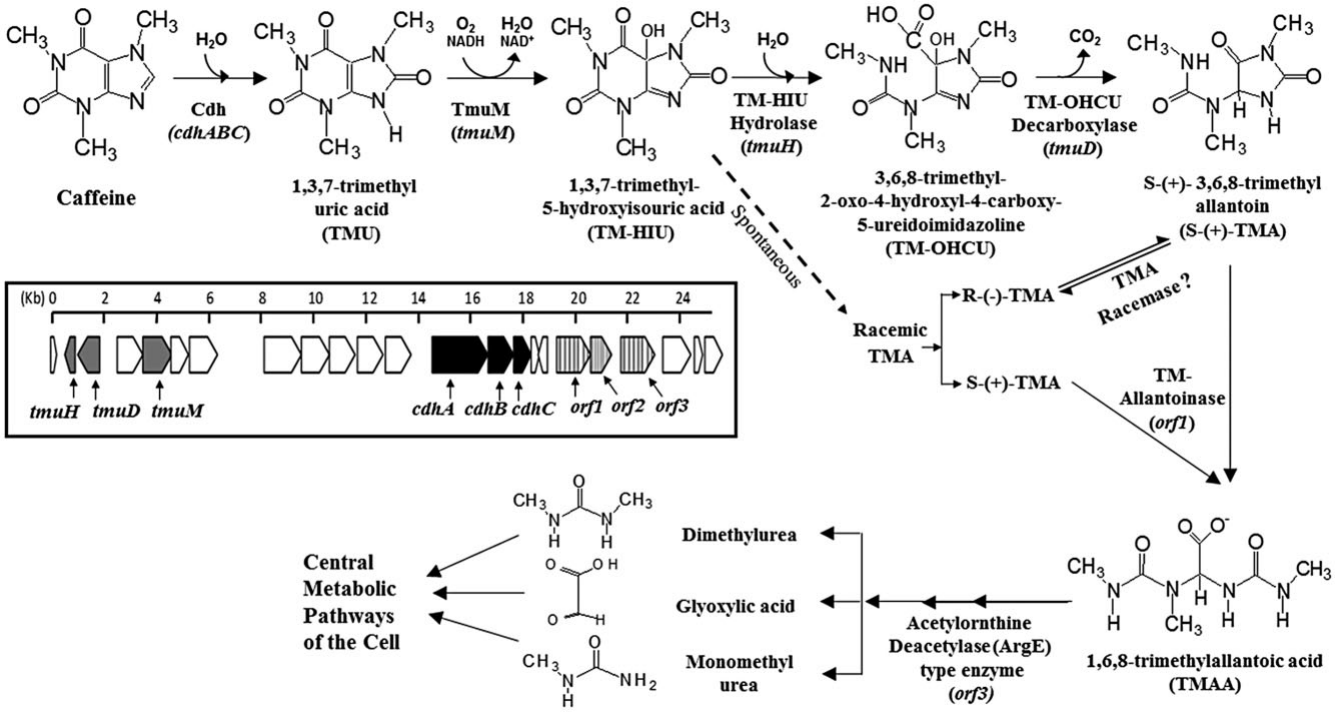
Proposed caffeine C-8 oxidation pathway and associated genes in *Pseudomonas* sp. CBB1. The solid arrows represent enzymatic steps, while the dashed arrow represents the spontaneous degradation of 1,3,7-trimethyl-5-hydroxyisouric acid (TM-HIU) to racemic TMA. Inset: map of the 25.2 kb caffeine gene cluster on the CBB1 genome containing the entire C-8 oxidation pathway genes. Cdh/*cdhABC* = trimeric caffeine dehydrogenase; TmuM/*tmuM* = trimethyluric acid monooxygenase; *tumH* = putative TM-HIU hydrolase; *tmuD* = putative TM-OHCU decarboxylase; *orf1* = putative trimethylallantoinase; *orf2*, *orf3* = putative genes responsible for catabolism of 1,6,8-trimethylallantoic acid to simpler metabolites such as dimethylurea, monomethylurea and glyoxylic acid.

C-8 oxidation involves the oxidation of caffeine to form 1,3,7-trimethyluric acid (TMU), which is further degraded by a pathway homologous to the uric acid metabolic pathway (Fig. 2). This pathway has been observed in both mixed cultures (Madyastha and Sridhar, 1998) and pure bacterial isolates (Mohapatra *et al*., 2006; Yu *et al*., 2008; Mohanty *et al*., 2012). 1,3,7-trimethyluric acid is further metabolized to sequentially form 1,3,7-trimethyl-5-hydroxyisouric acid (TM-HIU), 3,6,8-trimethyl-2-oxo-4-hydroxy-4-carboxy-5-ureidoimidazoline (TM-OHCU) and 3,6,8-trimethylallantoin (TMA) (Mohanty *et al*., 2012). Further degradation of TMA has not yet been fully characterized. However, it is believed that only S-(+)-TMA is formed enzymatically and its degradation proceeds through trimethylallantoic acid (TMAA) before being mineralized to glyoxylic acid, dimethylurea and monomethylurea (Madyastha and Sridhar, 1998; Mohanty, 2013). These latter compounds are then assumed to enter the central metabolic cycles of the bacterial cell.

## Genetic basis for caffeine degradation

Although bacterial degradation of caffeine has been studied for over 40 years, very little was discovered concerning the enzymes involved in the metabolism. Similarly, the genetics of bacterial caffeine metabolism were unknown. Recent work has revealed the nature of enzymes involved in both *N*-demethylation and C-8 oxidation pathways (Asano *et al*., 1994; Madyastha *et al*., 1999; Yamaoka-Yano and Mazzafera, 1999; Yu *et al*., 2008; 2009; Summers *et al*., 2011; Mohanty, *et al*., 2012). The gene sequences for these enzymes have also been elucidated, reported and deposited in the GenBank database (Mohanty *et al*., 2012; Summers *et al*., 2012; 2013). Currently, the only known bacterial genes responsible for caffeine metabolism are those that are directly responsible for catabolism. Other genes encoding proteins for caffeine uptake, chemotaxis and regulation of caffeine-degrading enzymes have not yet been reported.

### *N*-demethylation

Many of the earliest works on caffeine *N*-demethylase enzymes indicated that they are labile in nature and quickly lost activity during purification (Hohnloser *et al*., 1980; Glück and Lingens, 1988; Sideso *et al*., 2001; Beltran *et al*., 2006). Glück and Lingens (1988) reported partial purification of a 7-methylxanthine demethylase from *P. putida* WS that was not active towards any other methylxanthine. This enzyme lost activity within 12 h and could not be completely purified. Asano and colleagues (1994) observed two distinct *N*-demethylase fractions eluting from an ion-exchange chromatography column loaded with cell extracts of *P. putida* No. 352. One protein fraction was active towards caffeine, resulting in formation of theobromine. The second fraction converted theobromine to 7-methylxanthine. These results indicate that caffeine is *N*-demethylated by at least three methylxanthine-specific enzymes.

Recently, the five enzymes (NdmABCDE) that catalyse the entire caffeine *N*-demethylation pathway in *P. putida* CBB5 were purified and characterized (Summers *et al*., 2011; 2013). These same enzymes are also responsible for metabolism of theophylline in CBB5. In addition, the gene sequences of all five enzymes (*ndmABCDE*) were determined from a 13.2-kb CBB5 genomic DNA fragment (Fig. 1B) and deposited in the GenBank database (Summers *et al*., 2012; 2013). NdmA and NdmB are Rieske [2Fe-2S] monooxygenases with a non-heme iron at the active site (Summers *et al*., 2012). NdmA specifically removes the *N*_1_-methyl group from caffeine, paraxanthine, theophylline and 1-methylxanthine to form theobromine, 7-methylxanthine, 3-methylxanthine and xanthine respectively. Similarly, NdmB is an *N*_3_-specific demethylase, converting caffeine, theobromine, theophylline and 3-methylxanthine to paraxanthine, 7-methylxanthine, 1-methylxanthine and xanthine respectively.

Both NdmA and NdmB are entirely dependent upon NdmD (Fig. 1A), which is a partner reductase that transfers electrons from nicotinamide adenine dinucleotide (NADH) to power the reaction (Summers *et al*., 2011). NdmD is a redox-dense protein, containing two [2Fe-2S] clusters, one FMN binding domain and one NADH binding domain. When NdmD is expressed with NdmC and NdmE, the three proteins form a large protein complex that catalyses the *N*_7_-demethylation of 7-methylxanthine to xanthine (Summers *et al*., 2013). NdmC is also a non-heme iron monooxygenase but does not contain a Rieske [2Fe-2S] cluster, as do NdmA and NdmB. NdmE is a glutathione-*S*-transferase-like protein that is postulated to have a structural role in aligning the extra Rieske cluster found on NdmD with the NdmC subunit to catalyse the last *N*-demethylation step.

Among the Rieske oxygenase family, NdmCDE protein complex is very unique (Summers *et al*., 2013). The reductase, NdmD, is the only reported Rieske reductase that contains an extra [2Fe-2S] cluster. The NdmCDE protein complex is also the first reported instance in which the Rieske domain of the oxygenase is split off and fused to the reductase. Also, this was the first reported instance in which a glutathione-*S*-transferase-like protein is absolutely required for solubility and activity of a Rieske oxygenase. Interestingly, a blast search using the *ndmCDE* genes as queries identified an additional 18 organisms with homologous *ndmCDE* clusters coding for enzymes of unknown function (Summers *et al*., 2013). The *ndmCDE* genes clustered most closely with homologues from *Janthinobacter* sp. Marseille (accession no. NC_009659.1), *Klebsiella pneumoniae* subsp. *pneumoniae* WGLW2 (accession no. NZ_JH930420.1) and *Pseudomonas* sp. TJI-51 (accession no. NZ_AEWE01000207.1). Other bacteria containing *ndmCDE* homologues predominantly belong to the *Sinorhizobium* and *Mesorhizobium* genera. Although the functions of these homologues are unknown, the widespread dissemination of *ndmCDE* homologues indicates that similar caffeine-degrading enzymes may be found in bacteria other than those in the *Pseudomonas* genera.

Enzymes and genes homologous to NdmABCDE were also found in caffeine-grown *Pseudomonas* sp. CES cells (Yu *et al*., 2014). The sequences of these homologous enzymes revealed high similarity to the enzymes (80–90% identity) and genes (72–77% identity) in *P. putida* CBB5. The similarity between the caffeine-degrading enzymes in *P. putida* CBB5 and *Pseudomonas* sp. CES indicate that these *N*-demethylase enzymes may be conserved among many bacteria that metabolize caffeine *via N*-demethylation. The *ndmABCDE* genes in *P. putida* CBB5 and *Pseudomonas* sp. CES are encoded on the genomic DNA. In contrast, Dash and Gummadi (2006a) reported that *N*-demethylation in *Pseudomonas* sp. NCIM 5235 is encoded on a 12 kb plasmid, although the gene sequences have not yet been reported. Other caffeine *N*-demethylase genes may yet be discovered in the future, which will only increase our understanding of how bacteria respond to many other *N*-methylated compounds in the environment.

Flanking the *ndmABCDE* genes on the CBB5 chromosome are two genes homologous to those known to catalyse the conversion of formaldehyde to formic acid, *frmA* and *frmB* (Fig. 1B). Formaldehyde production during *N*-demethylation has been detected many times (Blecher and Lingens, 1977; Glück and Lingens, 1988; Summers *et al*., 2011; 2012; 2013). Thus, the genetic basis of caffeine *N*-demethylation, including the cellular utilization of formaldehyde produced as a by-product has been substantiated.

### C-8 oxidation

To date, three enzymes catalyzing the C-8 oxidation of caffeine have been purified and characterized. An 85-kDa caffeine oxidase was purified from a mixed culture of *Klebsiella* sp. and *Rhodococcus* sp. (Madyastha *et al*., 1999). Mohapatra and colleagues (2006) discovered a 65-kDa caffeine oxidase in *Alcaligenes* sp. Both caffeine oxidase enzymes displayed low activity towards theobromine, theophylline and a few other theobromine analogues. A heterotrimeric caffeine dehydrogenase (Cdh) enzyme was discovered in *Pseudomonas* sp. CBB1 (Yu *et al*., 2008), which catalysed C-8 oxidation of caffeine to form TMU (Fig. 2). This 158-kDa protein was a novel quinone-dependent oxidoreductase (EC 1.17.5.2) that exhibited no activity with NAD(P)^+^. The second step of the C-8 oxidation pathway, conversion of TMU to TM-HIU, was catalysed by a 43-kDa NADH-dependent trimethyluric acid monooxygenase (TmuM) also isolated from CBB1 (Mohanty *et al*., 2012). TM-HIU generated by TmuM was found to be unstable and spontaneously degraded to racemic TMA. However, the two step enzymatic transformation of TM-HIU is expected to yield S-(+)-TMA via TM-OHCU in biological systems, analogous to the uric acid metabolic pathway (Ramazzina *et al*., 2006).

In *Pseudomonas* sp. CBB1, several genes, including genes encoding Cdh and TmuM, have been identified in a 25.2-kb caffeine gene cluster of the CBB1 genome (Fig. 2 inset). A blastx-based sequence analysis of Cdh genes (*cdhABC*) revealed significant homology with other heterotrimeric enzymes such as xanthine dehydrogenase, hydratase/alcohol dehydrogenase, aldehyde oxidase and carbon monoxide dehydrogenase. The high similarity between *cdhABC* and their homologues further facilitated in associating these genes with their respective cofactor-binding subunits (Mohanty *et al*., 2012). Similarly, the *tmuM* gene showed significant homology with FAD (Flavin adenine dinucleotide)-containing aromatic-ring hydroxylases. In particular, *tmuM* showed a high degree of similarity with *hpxO* gene (encoding HpxO, a FAD-dependant uric acid oxidase) from *K. pneumoniae*. Further, a protein homology model of TmuM based on HpxO led to a better understanding of the changes in the active site pocket of this enzyme and its specificity towards methyluric acids (Mohanty *et al*., 2012).

Two other genes in this cluster, *tmuH* and *tmuD* (Fig. 2 inset), are proposed to encode putative enzymes (TM-HIU hydroxylase and TM-OHCU decarboxylase, respectively) of the C-8 oxidation pathway (Mohanty *et al*., 2012) based on their homology to enzymes of the uric acid metabolic pathway in *K. pneumoniae* (French and Ealick, 2010; 2011; Mohanty, 2013). The presence of these putative enzymes in CBB1 suggests enzymatic formation of S-(+)-TMA from TM-HIU via TM-OHCU in the C-8 oxidation pathway (Mohanty *et al*., 2012). *Klebsiella pneumoniae* also contains an allantoin racemase to covert (R)-allantoin, formed by spontaneous degradation of hydroxyisouric acid, to (S)-allantoin (French *et al*., 2011). Mohanty and colleagues (2012) proposed a similar conversion of (R)-(-)-TMA, formed by spontaneous degradation of TM-HIU, to (S)-(+)-TMA by a TMA racemase. However, none of the genes in the 25.2-kb CBB1 genetic cluster showed any homology to allantoin racemase (Mohanty, 2013). Thus, there yet remain undiscovered genes encoding enzymes active in the caffeine C-8 oxidation pathway.

Further degradation of S-(+)-TMA to glyoxylic acid, dimethylurea and monomethylurea, as suggested by Madyastha and Sridhar (1998), is poorly understood. Mohanty and colleagues (2012) have identified an open reading frame (*orf1*) in the 25.2-kb caffeine gene-cluster in the CBB1 genome, which encodes a putative trimethylallantoinase homologous to allantoinases that hydrolyse allantoin to allantoic acid (Fig. 2). This suggests that S-(+)-TMA is further hydrolysed into TMAA in the C-8 oxidation pathway. Subsequent formation of glyoxylic acid, dimethylurea and monomethylurea, which then enter the central metabolic pathway for total mineralization, may occur by hydrolysis of non-peptide C-N bonds of TMAA (Mohanty, 2013).

### C-8 oxidation of *N*-demethylated metabolites

There are several reports concerning the C-8 oxidation of *N*-demethylated metabolites. Woolfolk (1975) discovered a xanthine dehydrogenase capable of oxidizing xanthine, 1-methylanthine and 3-methylxanthine in *P. putida* 40. Yamaoka-Yano and Mazzafera (1999) reported that *P. putida* L contains a broad-specificity xanthine oxidase responsible for C-8 oxidation of theobromine, paraxanthine and 7-methylxanthine. The purified oxidase also oxidized xanthine, 3-methylxanthine and theophylline. *Pseudomonas putida* CBB5 also contains a broad-substrate xanthine dehydrogenase that was partially purified from cell extracts and found to be active towards theophylline, 1-methylxanthine, 3-methylxanthine and xanthine (Yu *et al*., 2009). In all cases, the methyluric acids formed from *N*-demethylated metabolites accumulate in the growth media and are not further degraded. In addition, none of the methyluric acids were utilized as sole carbon and nitrogen sources. Thus, the reason for their production is currently unknown, although it is likely that they are simply the result of a broad-specificity xanthine dehydrogenase that was partially purified from cell extracts of CBB5 (Yu *et al*., 2009). To our knowledge, these are the only three reports that describe the enzymes responsible for C-8 oxidation of *N*-demethylated metabolites in bacteria. Currently, it is unknown whether these enzymes are specific for *N*-demethylated metabolites or are simply the general xanthine dehydrogenase with broad specificity. No bacterial gene has been associated with the C-8 oxidation activity of *N*-demethylated caffeine metabolites to date.

## Applications of bacterial caffeine degradation

An understanding of the genes involved in bacterial caffeine degradation may open up several new biotechnological applications. Some of these include biological decaffeination of coffee, tea and caffeinated plant matter, environmental remediation of soils and waters with high caffeine concentrations, synthesis of alkylxanthines and alkyl uric acids for use as chemicals or pharmaceuticals and development of a rapid diagnostic test to detect caffeine and related methylxanthines.

### Bio-decaffeination

Bio-decaffeination of coffee and tea using whole microbial cells or enzymes has been discussed for a number of years (Kurtzman and Schwimmer, 1971; Sideso *et al*., 2001; Beltran *et al*., 2006; Gopishetty *et al*., 2012). *Pseudomonas putida* CBB5 can completely decaffeinate coffee and tea extracts, while *Pseudomonas* sp. CBB1 has also been used to decaffeinate tea extracts (Gopishetty *et al*., 2012). In terms of relative efficacy, strain CBB5 used the *N*-demethylation pathway to degrade a higher amount of caffeine in a shorter amount of time than did strain CBB1 through the C-8 oxidation pathway. An immobilized mixed culture of *Klebsiella* sp. and *Rhodococcus* sp. was also used to decaffeinate tea extract *via* C-8 oxidation under both batch and continuous processes (Summers *et al*., 2014). Overall, the *N*-demethylation pathway appears to be more efficient than C-8 oxidation for use in the microbial decaffeination of coffee. However, the use of bacterial cells for bio-decaffeination of beverages may not be feasible due to the potential for release of endotoxins.

Alternately, use of purified caffeine-degrading enzymes (either soluble or immobilized) may provide a viable alternative (Beltran *et al*., 2006; Gopishetty *et al*., 2012) to eliminate endotoxin problems. The *ndmABCDE* genes could be cloned into *Escherichia coli* for large-scale recombinant enzyme production in order to carry out bio-decaffeination of beverages. Another approach is to clone caffeine-degrading genes into *Saccharomyces cerevisiae* or another generally regarded as safe (GRAS) organism for whole-cell bio-decaffeination (Gopishetty *et al*., 2012), thus circumventing the endotoxin problem. Through this method, optimized genetic cassettes could be transformed into the GRAS organism, creating an enhanced caffeine-degrading strain.

A greater opportunity for microbial bio-decaffeination may be in the decaffeination of coffee and tea wastes. During processing of coffee, millions of metric tons of waste are generated each year (Brand *et al*., 2000). This waste has a high nutritional content, including a carbohydrate content of 45–60% (dry weight) (Summers *et al*., 2014). Unfortunately, a caffeine concentration in the waste greater than 1% makes it unsuitable as animal feed or as a biofuel feedstock. Treatment of coffee waste with caffeine-degrading microorganisms (either wild type or recombinant) may transform the waste into a valuable by-product, rather than a waste stream.

### Environmental remediation

There are many routes by which caffeine enters the environment, where it can exhibit toxic effects on the surrounding plants, insects and microbes (Nathanson, 1984; Waller, 1989). In coffee and tea fields, fallen leaves, stems and seeds decompose, releasing caffeine into the soil. Solid and liquid wastes from coffee and tea processing plants also contain high levels of caffeine, which enter soil and groundwater. The widespread use of caffeine in foods, beverages and pharmaceuticals lead to high levels of caffeine in human wastewater streams, as well. In fact, caffeine can be used as an anthropogenic marker for wastewater contamination in the environment (Buerge *et al*., 2003). In all of these cases, either wild-type or recombinant caffeine-degrading microorganisms may be of use in removing caffeine from contaminated environments.

### Chemical production

While caffeine is a relatively inexpensive molecule, many of the metabolites formed by both *N*-demethylation and C-8 oxidation of caffeine and their analogues are high-value chemicals. Many of these chemicals have great potential in the pharmaceutical and cosmetic industries. Uric acid and methyluric acids are antioxidants (Nishida, 1991; Schlotte *et al*., 1998), and 8-oxomethylxanthines may be used in treatments for obesity, skin cosmetics and anti-dandruff products (Simic and Jovanovic, 1989). Methylxanthines have been used as diuretics, bronchodilators, antioxidants and asthma control (Lee, 2000; Daly, 2003).

Most methylxanthines and methyluric acids are difficult to synthesize chemically because selective alkylation of each nitrogen atom is difficult to achieve (Taylor *et al*., 1961; Shamim *et al*., 1989; Gopishetty *et al*., 2012). The recent discovery of genes encoding bacterial caffeine-degrading enzymes may help to facilitate synthesis of these high-value chemicals. The *ndmABCDE* genes catalyse specific *N*-demethylation of alkylxanthines, which leave a specific methyl group open for chemical derivatization. Caffeine dehydrogenase from *Pseudomonas* sp. CBB1 can oxidize caffeine to TMU, and displays some activity towards other methylxanthines, as well.

Currently, there is only one report of methylxanthine production from caffeine using engineered cells (Summers *et al*., 2014). The genes *ndmA* and *ndmD* were cloned into *E. coli*, resulting in a bacterial strain that was able to effectively convert caffeine to theobromine. A second *E. coli* strain was constructed to convert theobromine to 7-methylxanthine using *ndmB* and *ndmD* genes. This preliminary report demonstrated the feasibility of specific methylxanthine production from caffeine.

### Diagnostics

**Fig 3:**
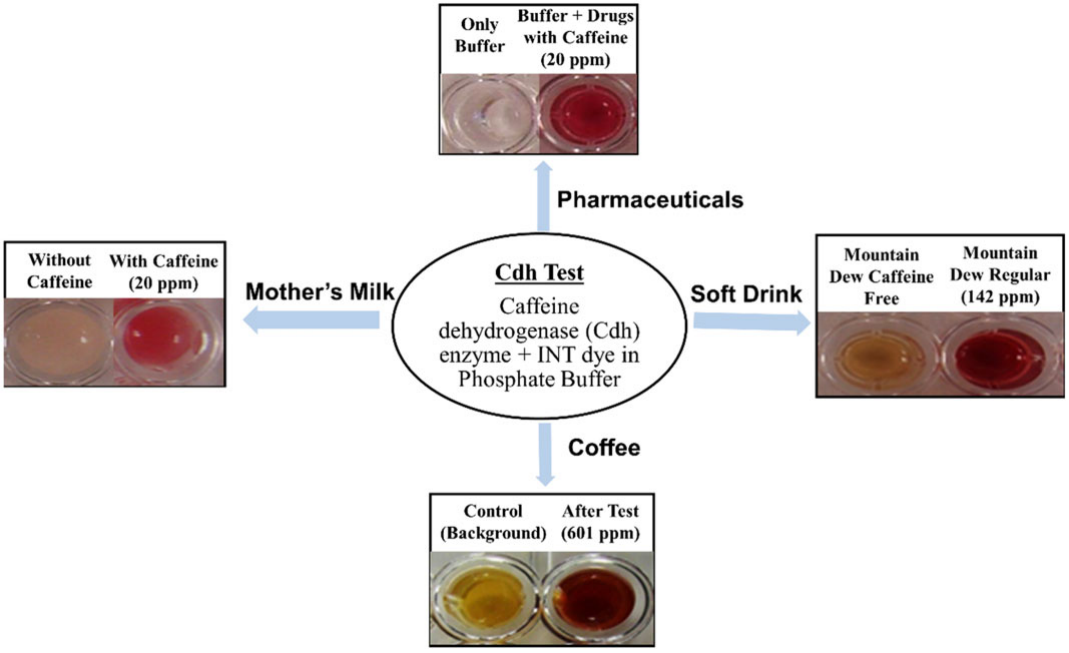
Caffeine dehydrogenase (Cdh)-based caffeine detection. Iodonitrotetrazolium chloride (INT) dye in the presence of Cdh results in shades of red to detect caffeine in test samples such as nursing mothers' milk (left, with 20 ppm caffeine), pharmaceuticals (top, tablets dissolved and diluted to 20 ppm caffeine), soft drinks (right) and brewed coffee (bottom).

Indiscriminate introduction of caffeine in food and beverages has led to growing concern among caffeine-sensitive consumers and the US Federal Drug Administration, giving rise to an ever-increasing demand for a suitable ‘in-home’ test to detect caffeine. Recently, a Cdh enzyme-based colorimetric test was developed. This test was rapid and sensitive enough to detect caffeine in beverages, including coffee, soft drinks and nursing mother's milk, within minutes (Mohanty *et al*., 2014). Based on the type of dye (electron acceptor) used, the test developed a bright colour upon exposure to caffeine even at 1–5 ppm level (Fig. 3). The test could successfully detect caffeine in samples with a wide range of pH and variations, with milk and sugar, or with other active pharmaceutical ingredients. Thus, this test is now deemed to be highly suitable for further development into an ‘in-home’ type strip-based test (Mohanty *et al*., 2014).

The *N*-demethylase genes were also used to addict *E. coli* to caffeine (Quandt *et al*., 2013), resulting in a whole-cell caffeine biosensor. Genes *ndmABCD* and an *ndmE* homologue were cloned into an *E. coli* guanine auxotroph. In order to grow, the engineered cells were required to convert caffeine to xanthine, which was further converted to guanine. This engineered *E. coli* strain was then used to accurately determine the caffeine concentration of various beverages. While impractical for an at-home caffeine diagnostic test, this *E. coli* strain could find use detecting caffeine in a laboratory setting or in environment samples.

## Conclusion

Research for over 40 years has uncovered two distinct caffeine metabolic pathways in bacteria: *N*-demethylation and C-8 oxidation. While there are a couple of reports on the enzymes involved in these processes, work was stalled due to lack of knowledge concerning the genetics of bacterial caffeine degradation. The recent discovery of bacterial genes responsible for metabolism of caffeine, by both *N*-demethylation and C-8 oxidation routes, opens numerous potential biotechnological applications. These novel genes and enzymes may be of great use in home diagnostic tests, remediation of caffeine-contaminated environments and production of chemicals, pharmaceuticals, animal feed and biofuels.

## Conflict of interest

None declared.
